# The Use of Prebiotic and Probiotic Interventions for Treating Gastrointestinal and Psychosocial Health Symptoms in Cancer Patients and Survivors: A Systematic Review

**DOI:** 10.1177/15347354211061733

**Published:** 2021-11-30

**Authors:** Julie M. Deleemans, Zen Gajtani, Mohamad Baydoun, Raylene A. Reimer, Katherine-Ann Piedalue, Linda E. Carlson

**Affiliations:** 1 University of Calgary, Calgary, AB, Canada

**Keywords:** prebiotics, probiotics, gastrointestinal, psychosocial health, quality of life, gut microbiota

## Abstract

**Background:**

Cancer treatments can cause significant gastrointestinal (GI) health issues, and negatively affect patient’s psychosocial health and quality of life (QOL). Novel, integrative strategies using prebiotics and probiotics have been explored for treating adverse cancer treatment-related side effects. We evaluated the current literature for interventions using prebiotics or probiotics specifically to treat GI and psychosocial health issues in cancer patients and survivors.

**Methods:**

Five databases (PubMed, MEDLINE (Ovid), CINHAL, PsychINFO, Web of Science) were searched for studies with prebiotic or probiotic interventions where GI and/or psychosocial health outcomes were measured in adult cancer patients and survivors, and published before September 12th 2021.

**Results:**

Twelve studies (N = 974 participants) meeting the inclusion criteria were identified (randomized controlled trials [n = 10], single-group pre-post studies [n = 2]). Ten studies were conducted with patients on active cancer treatment, and 2 studies treated patients after anti-cancer therapies. Three studies used prebiotics, 7 studies used probiotics, and 2 studies used a combination therapy. The most commonly used probiotic strains were from the *Lactobacillus* genus. There was minimal evidence for prebiotics to improve GI or psychosocial health. Probiotics were associated with significant improvements in abdominal pain (n = 2), gas/bloating (n = 2), and especially diarrhea (n = 5), and with improvements in anxiety (n = 1), depression (n = 1), fatigue (n = 1), and QOL (n = 2).

**Conclusions:**

Studies specifically examining effects of prebiotics and probiotics on GI and psychosocial health outcomes are scarce. Probiotic intervention may improve some GI symptoms in cancer patients, and QOL in survivors. Controlled trials that consistently include GI and psychosocial health outcomes are needed.

## Introduction

As gut microbiota research has rapidly expanded during recent decades, so too has our understanding of its role in disease states, such as cancer. The human gut microbiota is the community of microorganisms that exist within a single ecosystem and includes complex bacterial, archaeal, fungal, viral, and protozoan communities which help to maintain homeostasis and regulate major body systems, including the gastrointestinal tract and central nervous system (CNS).^1-3^ The gut microbiota has been implicated not only in the etiology of certain types of cancer, but also as a factor in disease progression, treatment efficacy, and anti-cancer treatment related toxicities.^1^ Focus has been directed toward harnessing the potential of the gut microbiota and its role in cancer for 2 broad purposes: (i) to enhance the therapeutic effects of anti-cancer treatments; and (ii) to ameliorate treatment-related side effects and improve patients’ treatment adherence and overall quality of life (QOL). Employing the gut microbiota to reduce treatment toxicities and improve patients’ experience is gaining traction within oncology care.

According to recent data, globally there were an estimated 19.3 million new cases of cancer in 2020, and 10 million cancer-related deaths.^4^ Following diagnosis and commencement of treatment, cancer patients may experience a variety of treatment-related toxicities and side effects, ranging from mild to severe. In some cases, these toxicities are so unbearable that patients may choose not to continue with treatments or need their therapeutic dose lowered. Additionally, many cancer treatments produce long-term effects, changes to the body lasting for months or years following treatment, including changes in cognition and mood.^5,6^ Cancer treatments are also shown to induce a dysbiotic state in the gut microbiota.^7,8^ Gut microbiota dysbiosis is broadly defined as change in the composition and function of the microbiota that is driven by environmental and host-related factors that disturb the microbial ecosystem such that its resistance and resilience capabilities are exceeded.^2^ Gut dysbiosis may result in augmented production of pro-inflammatory cytokines, which can alter the intestinal epithelial barrier and increase intestinal permeability (“leaky gut”). This compromises intestinal integrity and enhances translocation of bacteria and their products (eg, endotoxin) into the bloodstream, leading to further increases in systemic inflammation and greater vulnerability to gastrointestinal (GI) symptoms initiated by cancer treatments.^9,10^ Indeed, upwards of 70% of cancer patients receiving chemotherapy experience mucositis, a symptom of dysbiosis, which involves painful inflammation and ulceration of tissue along the alimentary tract^10,11^ and is associated with adverse changes in the gut microbiota.^12^

Research regarding chemotherapy and radiotherapy induced gut dysbiosis and treatment-related toxicities generally focuses on the physical side effects that result. Few studies have investigated the potential role of gut microbiota in the adverse psychological and cognitive side effects from cancer treatments. However, accumulating evidence from animal studies suggests that chemotherapy in particular is associated with behavioral disturbance, and changes in GI function and the gut microbiota. In mice, chemotherapy induced symptoms of sickness behaviors, include increased anxiety-like behavior, and increased levels of proinflammatory cytokine biomarkers, and gut microbiota alterations.^13^ Similarly, Loman et al^14^ found that paclitaxel-treated female mice showed increased fatigue and decreased cognitive performance concurrent with reduced microglia immunoreactivity, increased central levels of pro-inflammatory cytokine and chemokine gene expression, and gut microbiota and colonic morphological changes. Chemotherapy and subsequent changes in the gut microbiota have also been associated with chemotherapy-induced pain in mice.^15,16^

Evidence suggests dysregulation of the gut microbiota-brain axis, the multidirectional system that allows gut microbiota to communicate with the brain through various mechanisms, including neural, endocrine, immune, and metabolic pathways, and the vagus nerve,^17^ contributes to the adverse intestinal, psychological, and neurological problems experienced by patients following cancer treatment.^10,18,19^ This is not surprising considering what is now known about the nature of bi-directional communication between the brain and many of these same peripheral systems, specifically the nervous, immune, and endocrine systems, studied in the field of psychoneuroimmunology (PNI). Incorporating an awareness of the role of the gut microbiome in this intricate mind-body matrix is an acknowledgment of the further complexity of these inter-relationships. While knowledge regarding effects of chemotherapy on the gut microbiota continues to grow, there is a crucial need for research to focus specifically on the role of the gut microbiota in psychological and cognitive toxicities in cancer patients and survivors.

One of the most widely studied populations for the effects of prebiotics and probiotics on GI outcomes and to a lesser extent, mental health, is patients with irritable bowel syndrome (IBS). Prebiotics, such as chicory-root derived inulin and oligofructose, are substrates that are selectively utilized as fuel by host microorganisms and that confer a health benefit.^20^ Probiotics are live microorganisms which, when administered in adequate doses, confer a health benefit on the host,^21^ such as improving immune function and supporting the competitive exclusion of pathogens.^22,23^ Both prebiotics and probiotics have been studied extensively in IBS populations, and more recently novel “psychobiotics,” live bacteria that confer mental health benefits through interactions with the gut microbiota,^17,24^ have also been investigated. Importantly, IBS patients frequently experience GI symptoms such as chronic diarrhea and abdominal pain and bloating, with comorbid mental health conditions, such as anxiety and depression, occurring at rates significantly higher than those reported in healthy individuals.^25^

Studies have sought to utilize supplementation with prebiotics or probiotics to help alleviate GI and mental health symptoms in patients with IBS. There has been some consensus regarding probiotic treatment for GI issues, concluding that specific probiotics are beneficial for alleviating certain IBS symptoms, preventing antibiotic associated diarrhea, while demonstrating favorable safety.^26^ In a randomized trial, Huang et al^27^ examined the effects of probiotics combined with electroacupuncture versus standard treatment with antidepressant medication in depressed patients suffering from chronic diarrhea. Compared to patients receiving standard treatment, patients in the intervention group experienced significantly attenuated diarrhea, abdominal pain, sleep disturbance, and cognitive impairment. Additionally, significantly augmented serum levels of serotonin (5-HT) and brain derived neurotrophic factor (BDNF) were observed in the intervention group.^27^ Unfortunately, it is unknown whether the effect was due to the probiotic or the acupuncture. Nevertheless, given that upwards of 90% of serotonin is produced in the GI tract,^28,29^ this is an important finding and may suggest that the gut microbiota affect mood via mediating serotonin mechanisms in the gut microbiota-brain axis.

There are parallels between both physical and psychological symptoms in IBS patients and those experienced by cancer patients during and after treatment, a root cause or contributing factor of which may be dysbiosis of the gut microbiota. Novel research is therefore exploring the potential use of probiotic interventions with cancer patients and survivors. By co-administering health promoting bacteria during and/or after cancer treatments, gut health may be improved such that dysbiosis will be prevented or reversed and patients will benefit from reduced treatment toxicity side effects, improving health outcomes. In the oncology setting, probiotic treatment may benefit patients by helping to maintain intestinal microbiota balance, reduce potential pathogenic bacterial infection, improve bowel regularity, and restore homeostasis to the intestinal microbiota after antibiotic treatment.^30^

### Present Study

Cancer is a complex disease requiring integrative and novel approaches to enhance patient care and survivor QOL. Some studies investigating prebiotic or probiotic interventions in cancer cohorts include GI outcome measures, but few include patient reported outcomes for improvements in psychosocial health and QOL. Given the established link between gut microbiota and mental health,^17^ and the deleterious effects of anti-cancer treatments on patients’ QOL and psychosocial health, measuring these outcomes would greatly enhance such studies. Moreover, there is an established link between gut dysbiosis and certain neuropsychiatric illnesses,^17,31^ thus it is plausible that chemotherapy induced gut dysbiosis may impact the etiology and trajectory of GI and psychosocial health in cancer patients and survivors.^6,10^ The present systematic review evaluates the current literature on this topic, and highlights key implications and opportunities for future research.

## Methods

### Study Selection Criteria

To be eligible for inclusion in this systematic review, articles needed to (1) include an intervention using a prebiotic and/or probiotic, (2) be conducted in an adult cohort with cancer patients (ie, participants were currently on cancer treatment) or survivors (ie, participants had completed cancer treatments and the intervention took place after), (3) be published in a peer-reviewed journal in English, and (4) measure and report outcomes related to participant’s GI (ie, nausea, vomiting, constipation, diarrhea, stomach pain, cramps, gas, bloating, acid reflux, heartburn, gastroesophageal reflux disease (GERD), dyspepsia, and indigestion) and/or psychosocial health (ie, depression, anxiety, fatigue, pain, cognitive decline, social functioning, fear of cancer recurrence, emotional wellbeing, and mood). Interventions could include randomized controlled trials, and non-randomized controlled, pilot, or feasibility single-group trial designs. Book chapters, conference publications, dissertations, reviews, animal studies, and studies with only baseline data were not included.

### Search Strategy and Study Selection

A systematic search was conducted using 5 databases including PubMed, MEDLINE (Ovid), CINHAL, PsychINFO, and Web of Science for articles meeting the inclusion criteria and published before November 13th, 2020, and subsequently updated to include recent publications up to September 12th, 2021. Additional sources (eg, ClinicalTrials.gov; ASCO; ESMO; OAISter; Google; Google Scholar) were also searched. Key words related to prebiotics and probiotics included items such as prebiotic, probiotic, psychobiotic, fermented food, kombucha, sauerkraut, soy, yogurt, *Bifidobacterium*, Lactobacillus rhamnosus, *Lactobacillus*, and *Saccharomyces*, among others. The full list of search items can be found in Appendix A. This review was not registered.

**Figure 1.**
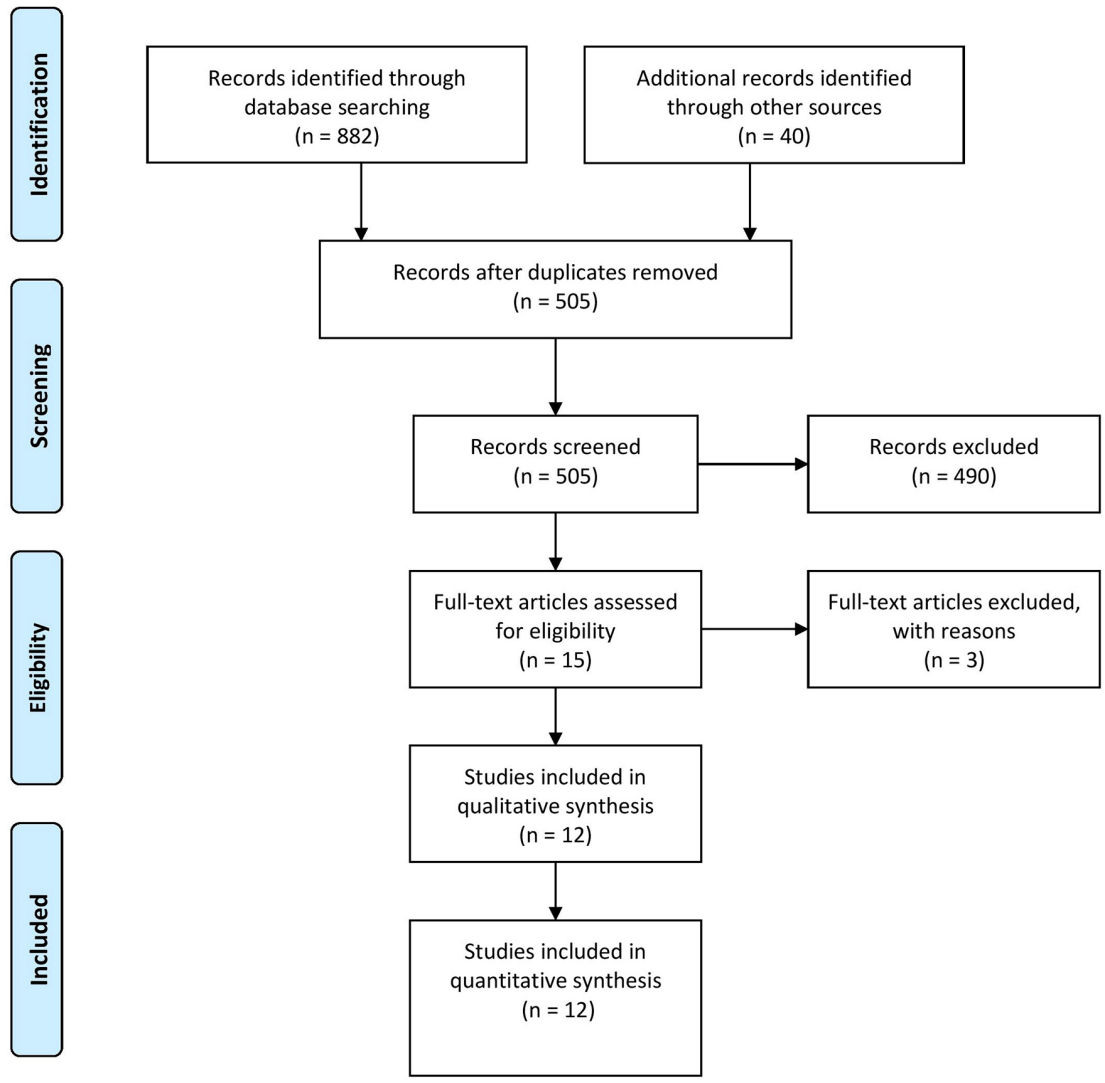
PRISMA process for data extraction.

The final results of the search process are illustrated in Figure 1. The PRISMA (preferred reporting items for systematic reviews and meta-analyses)^32^ process was applied during the completion of study review and selection. Two authors (JD and ZG or KAP) screened the titles and abstracts to determine eligibility. Any disagreements were resolved by discussion between the reviewing authors.

### Data Extraction

Fifteen full articles were reviewed by 2 authors (JD and ZG), and 3^33-35^ were subsequently excluded following closer examination due to not meeting inclusion criteria. In total, 12 full articles underwent data extraction which was completed by 2 authors (JD and ZG) using a locally designed template that summarized details of the participant characteristics, study design and setting, intervention type, dose and duration, and GI and psychosocial health outcome measures and results.

### Study Quality Assessment Procedures

The quality of the intervention trials described within the included articles were evaluated using the National Institutes of Health (NIH) Quality Assessment Tools.^36^ These tools are designed to assess different research designs for individual study quality, rather than the overall quality of the evidence. The NIH Assessment Tool for Controlled Trials was used to evaluate the controlled intervention studies, which we rated as “poor,” “fair,” or “good” according to criteria such as randomization, groups’ similarity at baseline, and blinding. The NIH Quality Assessment Tool for Uncontrolled Pre-Post Studies which includes criteria such as study objectives, eligibility criteria, and sample size was also used as appropriate. Studies were rated as “poor” if 33% or fewer of the criteria were met, “fair” if 34% to 67% of criteria were met, and “good” if 68% or more of the criteria were met. Two authors (JD and ZG) performed parallel quality assessments of the articles included, resolving any disagreements via discussion.

## Results

The search yielded a total of 922 citations. After removing duplicates, 505 articles remained and were screened for inclusion according to the eligibility criteria. After full-text screening, 12 articles^37-48^ qualified for inclusion in this systematic review. The characteristics of each study are presented in Table 1.

**Table 1.** Characteristics of Included Studies.

Reference	Sample	Study design	Intervention	Measures	Key findings and conclusions
De Loera-Rodriguez et al,^37^ Mexico	70 patients with cervical cancer, on pelvic radiotherapy and chemotherapy, mean age 49 years, 100% female	Randomized, double-blind controlled trial	*Treatment*: **Synbiotic** biogel with 1 × 10^7^ CFU/g *Lactobacillus acidophilus* NCFM, *Bifidobacterium lactis* Bi-07, 1 × 10^6^ CFU/g biogel, and blue agave inulin 3× daily, or placebo *Duration*: 7 weeks	(i) Bristol stool form scale (BSFS) (ii) National Institute of Cancerology (INC) of the United States scale for nausea and vomiting	• Significant decrease in vomiting frequency and intensity in treatment group vs controls (*P* < .001). • No significant effects on stool consistency or nausea. • In patients with cervical cancer, **synbiotic treatment significantly decreased vomiting frequency and intensity**.
Demers et al,^38^ Canada	229 patients with prostate, endometrial, cervical, or rectal cancer, on pelvic radiotherapy treatment, mean age 61 years, 96.7% male	Randomized, double-blind controlled trial	*Treatment*: **Probiotic** “Bifilact” *Lactobacillus acidophilus* LAC-361 and *Bifidobacterium longum* BB-536 at doses of either (i) 1.3 billion CFU, 2× daily, or (ii) 10 billion CFU, 3× daily, or (iii) placebo *Duration*: 11 weeks	(i) European Organization for Research and Treatment of Cancer (EORTC-QLQ-C30); (ii) World Health Organization (WHO) toxicity criteria for diarrhea severity; (iii) National Cancer Institute (NCI) scale V3.0 for Abdominal Pain	• Patients operated on and treated with the standard dose of Bifilact had less grade 4 diarrhea (97%) vs placebo group (*P* = .03). • In Bifilact standard-dose group, 35% of patients were without moderate to severe diarrheal symptoms vs. placebo (17%) after 60 days (*P* = .04). • For patients who had surgery the standard-dose probiotics reduced incidence of diarrhea of all grades (*P* = .05). • Probiotic intake did not affect patient QoL. • No significant effects on abdominal pain. • At the end of treatment, **Radiation induced** diarrhea **grades 2-4 was significantly reduced by the standard dose of Bifilact**.
Lee et al,^39^ Korea	60 survivors of colorectal cancer who completed radiotherapy and chemotherapy within the last 2 years, mean age 56 years, 58.3% male	Randomized, double-blind controlled trial	*Treatment*: **Probiotic** “Lacidofil” L. *rhamnosus* R0011, L. acidophilus R0052, 2 × 10^9^ CFU, 2× daily, or placebo *Duration*: 12 weeks	(i) ROME III criteria for bowel symptoms (ii) Functional Assessment of Cancer Therapy (FACT-V4) for cancer-related QoL; subscales: FACT-G, FACT-C, FACT-F, FACT-NTX scales (iii) Patient Health Questionnaire (PHQ)-9 for mental health, anxiety and depression domains	• Probiotics significantly decreased the proportion of patients with irritable bowel symptoms (*P* = .03) • Probiotics significantly improved colorectal cancer-related QoL (FACT-C) (*P* = .04), cancer-related fatigue (FACT-F) (*P* = .02), and anxiety and depression (PHQ-9) (*P* = .01) after 12 weeks • In colorectal cancer survivors, **probiotics improved bowel symptoms and quality of life**
Linn et al,^40^ Myanmar	54 cervical cancer patients completing pelvic radiotherapy and chemotherapy (76%), mean age 55 years, 100% female	Randomized, double-blind controlled trial	*Treatment*: **Probiotic** “Biogurt” capsule with 300 g functional yogurt containing 1.75 billion lyophilized live L. acidophilus LA-5 and *B. animalis subsp. Lactis* BB-12, 3× daily, or placebo *Duration*: 5 weeks	(i) Common Terminology Criteria for Adverse Events (CTCAE V-4.0) for radiation induced diarrhea and abdominal pain severity	• Significant reduction in radiation induced diarrhea for probiotic group vs. controls (*P* = .025) • Probiotics significantly attenuated mild-to-moderate (*P* = .025) and severe (*P* = .05) diarrhea • Probiotics significantly reduced grade 2 abdominal pain severity and duration of abdominal pain episodes (P’s = .000) • In cervical cancer patients, **probiotics significantly reduced the incidence and severity of radiation induced** diarrhea **and abdominal pain**
Liu and Huang,^46^ China	100 patients with gastric, colorectal, lymphoma, lung, or other unspecified types of cancer, all receiving chemotherapy, mean age 61 years, 68% male	Single group, pre-post trial	*Treatment*: **Probiotic** “Siliankang” *Bifidobacterium tetragenous* tablets with Bifidobacterium infantis, Lactobacillus acidophilus, Enterococcus faecalis and Bacillus cereus, 3× daily or standard care chemotherapy with no probiotics *Duration*: 4 weeks	(i) Rome II Chronic functional constipation scale (ii) Wexner constipation scale	• Significant **improvement in constipation for 96% of patients in probiotic group**, vs 32% in control group (*P* < .05)
Ohigashi et al,^41^ Japan	63 survivors of colon and rectal cancers treated with surgery and pelvic radiotherapy, mean time off treatment 6.5 years, mean age 65 years, 57% male	Single group, pre-post trial	*Treatment*: **Probiotic** “The Guard” tablets containing *Bacillus natto*, Lactobacillus acidophilus, 3 tablets, 3× daily. Total daily dose was 10 mg of *B. natto* and 30 mg of L. acidophilus *Duration*: 12 weeks	(i) Medical Outcomes Study Short-Form 36-Item Health Survey (SF-36, V2) for QoL (ii) European Organization for Research and Treatment of Cancer Quality of Life Questionnaire C30 (QLQ-C30, V3) for QoL (iii) Wexner Incontinence scale for functional outcomes	• In the left-side colon cancer group, probiotics significantly improved emotional function (*P* < .01) • Probiotics significantly improved global QoL for the right-side colon and rectal cancer groups (*P*’s < .05) • Probiotics significantly improved constipation for the left-side colon and rectal group (P’s < .01) • Probiotics significantly reduced diarrhea in rectal cancer group (*P* = .01) • In left-side colon group, probiotics significantly attenuated defecation frequency (*P* < .05) • In right-side colon group, probiotics significantly reduced defecation frequency and frequency of incomplete defecation (*P* < .05) • For patients who receive rectal resection or right-side colectomy, **probiotics may help improve QoL and functional outcomes**
Osterlund et al,^47^ Finland	150 colorectal cancer patients on chemotherapy (100%), plus radiotherapy (37%), median age 60 years, 51% male	Randomized, controlled trial	*Treatment*: **Synbiotic** “Gefilus” Lactobacillus rhamnosus GG 1-2 10^10^ CFU 2× daily, Gaur gum (prebiotic) 11×g, 1× daily, on chemotherapy cycle days 7-14, for 8 days per month, or no supplement *Duration*: 24 weeks	(i) Common Toxicity Criteria of the NCI of Canada scale (V2)	• Grade 3-4 diarrhea significantly lower in patients receiving probiotics (*P* = .027) • Probiotic treatment significantly reduced abdominal pain (*P* = .025) • 21% of patients receiving probiotics had chemotherapy-dose reductions due to bowel toxicity vs 47% in control patients (*P* < .00) • No significant effect on overall chemotherapy toxicity, frequency of stomatitis, or neutropenia • No significant effects of gaur gum fiber supplementation • **Probiotics significantly improved diarrhea, abdominal pain, and the need for chemotherapy dose reductions**
Rosli et al,^42^ Malaysia	30 patients with endometrial, cervical, colon, rectal, or prostate cancer receiving pelvic radiotherapy, mean age 57 years, 27% male	Randomized, double-blind controlled trial	*Treatment*: **Prebiotic** Partially hydrolyzed guar gum (PHGG; natural water-soluble fiber source) 2× daily, or placebo *Duration*: 4 weeks	(i) Bristol Stool chart for diarrhea incidence (ii) The European Organization for Research and Treatment of Cancer Care Quality of Life Questionnaire (EORTC-QLQ-C30; V 3.0) for QoL	• Intervention significantly reduced diarrhea frequency by day 45 (*P* < .05) • No significant difference for QoL scores • **Prebiotic PHGG supplementation reduced the frequency of** diarrhea **following pelvic radiation treatments**
Sasidharan et al,^43^ India	104 cervical cancer patients receiving pelvic radiation and chemotherapy, mean age 49, 100% female	Randomized, placebo controlled trial	*Treatment*: **Prebiotic** 30 g of HAMS Hylon VII resistant starch 2× daily, or placebo *Duration*: 6 weeks	(i) Common Toxicity Criteria (V3.0), and (ii) Radiation Therapy Oncology Group toxicity scale for diarrhea and proctitis severity	• No significant effects of the intervention on diarrhea or proctitis symptoms • No significant benefit of administering resistant starch to patients receiving pelvic radiotherapy
Shao et al,^44^ China	46 patients with abdominal or pelvic cancer completing radiotherapy or within 3 weeks post-radiotherapy, mean age 60 years, 48% male	Randomized, controlled trial	*Treatment*: **Probiotic** “Golden Bifid” with *Bifidobacterium, lactobacillus* and Streptococcus thermophilus, 0.35 g/capsule, *Bifidobacterium adolescent* 0.5 Billion CFU, 3 tabs 3× daily, and Glutamine enteric capsule (0.25 g/capsule) 2 capsules 2× daily, and Fish oil capsule (1200 mg/capsule), 1 capsule 3× daily, or Peptisorb *Duration*: 2 weeks	Not specified	• Occurrence rate of abdominal pain, flatulence and diarrhea on 7 and 14 days post-treatment was significantly lower in treatment group vs controls (*P* < .05) • In patients with **acute radiation enteritis, enteral nutrition may improve bowel function**
Wierdsma et al,^45^ The Netherlands	32 head and neck cancer survivors, mean age 58 years, 75% male	Prospective, randomized, double-blind pilot trial	*Treatment*: **Prebiotic** Standard no residue tube feeding with: (i) “Osmolite” non-fructooligosaccharides (FOS) group with no fiber, (ii) FOS and fiber-enriched with “Jevity FOS” 10.6 g fiber, 7 g FOS per liter, or (iii) control (standard western diet) *Duration*: 6 weeks	(i) Gastrointestinal Quality of Life Index (GIQLI) questionnaire for gastrointestinal QoL	• GIQOL in the FOS group remained stable, but in the non-FOS group decreased significantly during the 6 weeks intervention (*P* = .027). • **Prebiotic tube feeding with FOS may improve patient QoL byway of modulating the gut microbiota**, specifically increasing *Bifidobacteria*.
Yoon et al,^48^ Korea	36 rectal cancer patients, stages 1-3, receiving surgery for lower anterior resection and ileostomy reversal, plus preoperative chemoradiation (69.4%) completed 5 months prior. Mean age of 60 years, 64% male	Randomized, double-blind, placebo-controlled pilot trial	*Treatment*: **Probiotic** Lactobacillus plantarum CJLP243 (KCCM11045P), isolated from kimchi, 1× daily at a dose of 1 × 1010 or placebo, beginning 1 day after surgery *Duration*: 3 weeks	(i) Memorial Sloan Kettering Cancer Centre (MSKCC) Bowel Function Index (BFI) and LARS scores (ii) EORTC: QLQ-C30, QLQ-CR29	• No significant effects for GI or QOL outcomes

### Study and Participant Characteristics

Of the studies included, 10 were randomized controlled trials^37-40,42-45,47,48^ and 2 were a pre-post single-group design.^41,46^ As seen in Table 1, the geographical locations of the included studies varied with each of the included studies having been conducted in a different country, with the exception of 2 that were conducted in Korea^39,48^and 2 others conducted in China.^44,46^

A total of 974 participants were included across all 12 studies, with sample sizes ranging from 30 to 229 adult participants. The mean age of participants was 58 years, and sex distributions varied with 3 studies focusing exclusively on female cancer patients currently receiving treatment,^37,40,43^ and 9 studies ranging from 27% to 97.7% of the participants being male cancer patients on active treatment^38,42,44-48^ or survivors who had completed treatment.^39,41^ For the 2 studies that focused on cancer survivors who had completed treatment, the mean time off treatment ranged from 2^39^ to 6.5 years.^41^ In the study by Yoon et al^48^ 69.4% of participants had completed chemoradiation 5 months prior, but 100% of participants were receiving cancer-related surgery at the time of intervention and therefore considered to still be on active treatment. All other studies focused on patients currently receiving active treatments for cancer, primarily pelvic radiotherapy with or without chemotherapy,^37,38,40,42-44^ chemotherapy,^46,47^ and 1 study with patients receiving treatment for head and neck cancer.^45^ Three studies focused exclusively on cervical cancer patients,^37,40,43^ 1 study each focused on colorectal cancer survivors,^39^ colorectal patients,^47^ rectal patients,^48^ head and neck cancer patients,^45^ patients with diverse cancer diagnoses,^46^ and 4 studies involved patients or survivors with various cancers of the pelvic region, including prostate, endometrial, cervical, colon, or rectal cancers.^38,41,42,44^ Cancer stage ranging from 1 to 4 was reported in 6 studies.^37,39,40,42,43,47,48^ Only 2 studies reported on demographic factors including ethnicity, marital status, occupation, and education.^37,42^

### Prebiotic and Probiotic Intervention Characteristics

Seven studies involved interventions examining the effects of probiotics,^38-41,44,46,48^ 3 investigated prebiotics,^42,43,45^ and 2 studies used a combination of prebiotics and probiotics, referred to as a synbiotic.^37,47^ As seen in Table 1, treatment duration varied from 2 to 24 weeks. For probiotics, 3 studies administered treatment for 5 weeks or less,^40,44,48^ 3 studies treated participants for 11 to 12 weeks,^38,39,41^and 1 study for 24 weeks.^47^ Participants receiving prebiotic interventions were treated for 4^42^ and 6^43,45^ weeks. Participants receiving the synbiotic therapy were treated for 7 weeks^37^ and 8 days per month for 24 weeks.^47^ Intervention treatments were self-administered orally in all studies except for 1, which involved tube fed head and neck cancer patients.^45^ Consumption was completed in the participants’ homes, as supplementation was to be completed daily.

As seen in Table 1, among interventions using probiotics^38-41,44,46,48^ or synbiotics,^37,47^ various probiotic strains and doses were used. The most consistently used probiotic strains belonged to the *Lactobacillus* genus (100%),^37-41,44,46-48^ followed by those from the *Bifidobacterium* genus (56%).^37,38,40,44,46^ Doses and treatment duration varied from one study to the next, with minimal consistency observed between studies. Three studies investigated the effects of prebiotic interventions.^42,43,45^ Similar to probiotic interventions, there was limited consistency between prebiotic studies with respect to the type and dose of prebiotic used.

### Intervention Effects on Gastrointestinal and Mental Health Outcomes

**Figure 2.**
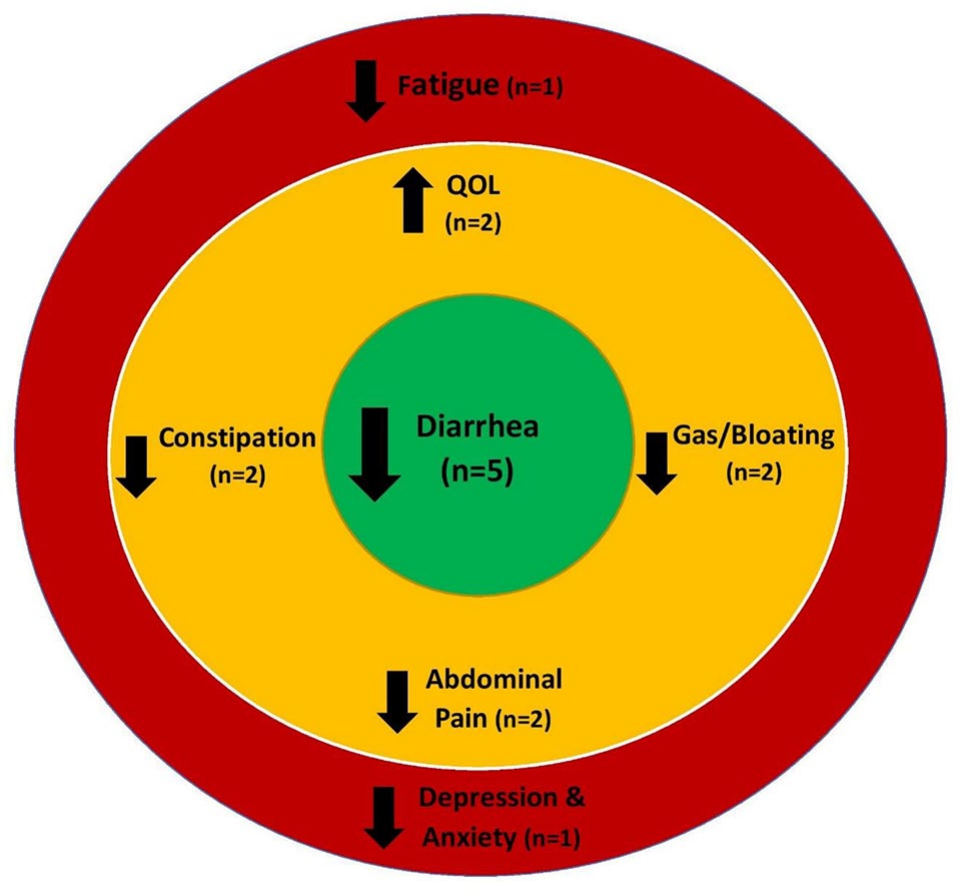
Summary of effects of probiotics on GI and psychosocial symptoms.

As seen in Figure 2, probiotic treatment was found to significantly improve several GI and some psychosocial health outcomes (ie, QOL) in cancer patients and survivors. In total, 11 studies investigated the effects of a prebiotic,^42,43^ probiotic,^38-41,44,46,48^ or synbiotic^37,47^ treatment on GI outcomes. As seen in Table 1, a variety of measures were used to evaluate GI symptoms. The most common GI symptom outcomes measured were diarrhea, constipation, and abdominal pain. Among studies investigating probiotic treatments, 4 found a significant decrease in diarrhea frequency and/or severity,^38,40,41,44^ 2 observed a significant reduction in irritable bowel symptoms such as gas and bloating,^39,44^ 2 indicated significant reductions in the severity and duration of abdominal pain,^40,44^ and 2 showed reduced constipation.^41,46^

Two studies investigated the effects of a synbiotic treatment. De Loera-Rodriguez et al^37^ found a significant reduction in vomiting frequency and intensity following the intervention. Osterlund et al^47^ found that patients who received the Lactobacillus rhamnosus *GG* (LGG) probiotic had significant reductions in moderate to severe diarrhea with only 22% reporting problems in the intervention group, compared to 37% in the control group. LLG treated patients also reported less abdominal discomfort, required less hospital care, and experienced fewer reductions in chemotherapy dose due to bowel toxicity.^47^ Notably, significant effects were only found for the probiotic treatment in the study by Osterlund et al,^47^ and prebiotic guar gum fiber was not found to have a significant effect on patient’s GI outcomes. Prebiotic interventions were found to be less effective in treating GI symptoms, as one study showed no significant effects of treatment,^43^ and one other study only observed a significant reduction in diarrhea following the intervention.^42^ Collectively, these results suggest that probiotics, particularly those with strains from the *Lactobacillus* genus, are most effective in supporting GI health in cancer patients and survivors.

Six studies examined the effects of prebiotics or probiotics on psychosocial health related outcomes in cancer patients and survivors.^38,39,41,42,45,48^ Table 1 illustrates the measurement tools used to quantify these outcomes. QOL was measured in 6 studies,^38,39,41,42,45,48^ and only 1 study measured fatigue, anxiety, and depression.^39^ Importantly, significant improvements in QOL were found only in survivors of cancer who had completed their anti-cancer therapies and who received a probiotic treatment.^39,41^ Among patients currently receiving cancer treatments, no improvements in QOL were observed following probiotic intervention.^38,48^ Prebiotic treatment was only found to maintain QOL scores from pre- to post-intervention in head and neck cancer patients,^45^ but was not found to significantly improve QOL among patients receiving pelvic radiotherapy.^42,43^ Notably, Lee et al^39^ was the only study to measure the effects of probiotics on other psychosocial health-related outcomes in cancer survivors and found that after the 12 week probiotic intervention cancer-related fatigue, anxiety, and depression all significantly improved. Although few studies have examined the effects of prebiotics and probiotics on psychosocial health related outcomes in cancer cohorts, based on these findings there is some suggestion that probiotics may be useful for cancer survivors who have completed treatments.

### Study Quality Assessment

Details of the quality assessment for the included studies are seen in Table 2. Ten of the included studies utilized a randomized controlled trial design.^37-40,42-45,47,48^ Two studies utilized a pre-post, single-group design.^41,46^ Despite small sample sizes in some studies, 7 of these studies^37-40,43,47,48^ were methodologically sound resulting in scores ranging from 100% to 79% and a rating of “good.” Two of the RCTs^42,45^, one pre-post single group study^41^ only met some of the criteria and thus received a rating of “fair.” One RCT^44^ and one pre-post single group study^46^ had numerous methodological issues and met minimal assessment criteria and were therefore rated as “poor.”

**Table 2.** Quality Assessment of Included Studies Based on the National Institutes of Health (NIH) Quality Assessment Tools.

Reference	No. of Criteria Met (n)	Score (%)	Rating	(#) Items not met or unspecified
*RCT studies—rated out of 14 items*				
De Loera-Rodríguez et al,^37^ Mexico	13	93	Good	(13) analyses prespecified
Demers et al,^38^ Canada	14	100	Good	
Lee et al,^39^ Korea	13	93	Good	(3) treatment allocation concealment
Linn et al,^40^ Myanmar	13	93	Good	(12) sample size reporting and 80% power analysis
Osterlund et al,^47^ Finland	11	79	Good	(4-5) Blinding procedures, (8) drop out rate
Rosli et al,^42^ Malaysia	10	64	Fair	(3) treatment allocation concealment, (6) group similarity at baseline, (7) drop-out rate, (9) intervention adherence
Sasidharan et al,^43^ India	13	93	Good	(6) Group similarity at baseline
Shao et al,^44^ China	3	21	Poor	Items 2-10 unspecified, (11) outcome measures not listed/described, (12) sample size reporting and 80% power analysis
Wierdsma et al,^45^ The Netherlands	7	50	Fair	(2) Adequate randomization, (3) treatment allocation concealment, (8) drop-out rate, (9) intervention adherence, (11) outcome measures, (12) sample size reporting and 80% power analysis, (13) analyses prespecified
Yoon et al,^48^ Korea	13	93	Good	(12) sample size reporting and 80% power analysis
*Pre-Post Single Group Trials—rated out of 12 items*				
Liu and Huang,^46^ China	4	33	Poor	(1) study objective defined, (4) enrolled all eligible participants, (6) methods clearly described, (7-9) measures, blinding, loss to follow-up, (11) interrupted time series design, (12) individual level data
Ohigashi et al,^41^ Japan	8	67	Fair	(4) enrolled all eligible participants, (5) sufficiently large sample size, (9) loss to follow-up, (11) interrupted time series design

## Discussion

A systematic review of the literature was conducted for studies that have investigated the effects of a prebiotic or probiotic intervention on GI and psychosocial health-related outcomes in adult cancer patients and survivors. Twelve studies met the inclusion criteria.^37-48^ Prebiotic and probiotic intervention characteristics varied with limited consistency observed in probiotic strains, doses, or treatment duration. The most consistently used probiotic strains belonged to the *Lactobacillus* genus. Most of the studies measured GI outcomes, such as diarrhea and abdominal pain but only 6 studies measured psychosocial health related outcomes,^38,39,41,42,45,48^ primarily QOL, and only the study by Lee et al^39^ measured fatigue, anxiety, and depression. Overall, probiotic interventions appeared to be most effective for alleviating GI symptoms, especially diarrhea, abdominal pain, and gas and bloating. Interestingly, significant improvements in QOL were only observed in survivors of cancer who had completed their anti-cancer therapies and who received probiotic treatment,^39,41^ but not in patients currently undergoing cancer treatments.^38,48^

### Implications for Cancer Patients and Survivors

A growing body of research supports the use of probiotic treatments for conditions such as IBS,^26^ to prevent infections and certain treatment toxicities in children undergoing treatments for cancer,^9^ and to a degree, also for some mental health conditions, such as depression.^27,49,50^ Gut microbiota dysbiosis may exacerbate or initiate hypothalamic-pituitary-adrenal (HPA) axis dysregulation. Evidence of this has been found in patients with major depressive disorder and anxiety disorders, which are often characterized by abnormal HPA axis function, responses to stress, and distinct differences in the gut microbiota compared to healthy people.^51,52^ Additionally, patients with IBS frequently present with comorbid depression and anxiety, in addition to their GI issues of which gut dysbiosis is a hallmark.^17^ Treatment with prebiotics and/or probiotics has been shown to attenuate GI symptoms, which is often accompanied by a reduction in anxiety and depressive symptoms.^53^ However, a paucity of research exists examining the effects of prebiotics and probiotics on GI and psychosocial health outcomes in adult cancer patients and survivors and it is evident that more research is needed. Based on the current evidence, supplementing one’s diet with probiotics during and/or after cancer treatments may help to alleviate certain GI symptoms, such as diarrhea and abdominal pain. This finding is consistent with other studies in patients with IBS.^26,27^

Few studies have examined the effects of prebiotics or probiotics on psychosocial health related outcomes within cancer cohorts. However, studies with other patient cohorts have shown that probiotic supplementation may help to alleviate some symptoms of mental health conditions, such as anxiety and depression.^27,49,50^ Importantly, of the studies reported here that did measure QOL, significant improvements were only found for cancer survivors who had completed their cancer treatments and received a probiotic,^39,41^ but not for patients currently receiving probiotics while on active treatment.^38,48^ This could be a reflection of the usual pattern of decreasing QOL over the course of cancer treatments, which the probiotic treatment may not have been able to attenuate. Notably, Wierdsma et al^45^ did find that following prebiotic intervention QOL remained stable in head and neck cancer patients in the intervention group while QOL deteriorated in the usual care control group. In this case, no decrease in QOL can still be interpreted as a success. The findings regarding QOL and patient treatment status (ie, active vs post) may also suggest that during the acute phase of treatment, when anti-cancer therapies are inflicting insult to organ systems and the gut microbiota, probiotic supplementation may have little impact on supporting QOL, although it can still help to alleviate GI symptoms. However, following cancer treatments, when the body begins to recover, supplementing one’s diet with probiotics may help to recolonize the gut microbiota with beneficial species. This, in turn, may support the microbiota-gut-brain axis and immune function, helping to alleviate the expression of inflammatory-related sickness behaviors,^54^ such as fatigue, anxiety, and depression, and to improve QOL.

Previous studies have shown associations between the gut microbiota and psychoneurological symptom clusters, such as fear of cancer recurrence, fatigue, and anxiety.^55-57^ For instance, in women with breast cancer who had previously completed chemotherapy, lower alpha diversity and relative abundance of *Firmicutes* and higher relative abundance of *Bacteroidetes* was significantly associated with fear of cancer recurrence compared to women who had not received chemotherapy.^56^ Additionally, Wang et al^58^ found a reduction in postoperative cognitive impairment and plasma IL-6 and cortisol levels following treatment with oral probiotics, compared to a placebo control group, in elderly patients following elective orthopedic or colorectal cancer surgery. Microbiota-based therapies hold promise for novel treatments to prevent or reduce treatment-related side effects and improve patients QOL. While much research is needed before definitive recommendations can be made, supplementing one’s diet with probiotics may help to improve adverse GI symptoms in patients on active treatment and survivors, and could potentially also support QOL and psychosocial health in survivors.

### Potential Mechanisms

Evolution in experimental methods has allowed for a better understanding of the gut microbiota-related factors that contribute to cancer and anti-cancer therapy related side effects, including adverse GI symptoms. Chemotherapy, immunotherapy, hormone therapies, and radiotherapy are all shown to affect the gut microbiota in cancer patients.^28^ For instance, chemotherapy impacts the immune system, local GI inflammation, and gut barrier function, while gut microbiota are shown to metabolize xenobiotic chemotherapy drugs and influence the response to immune checkpoint inhibitors in immunotherapy.^28^ However, to date, most evidence regarding mechanisms of prebiotics and probiotics on the gut microbiota and cancer treatment-related toxicities originates from animal studies. Certain bacterial species, typically administered as probiotics, have been demonstrated to have anti-tumor effects by supporting microbiota and immune modulation, enhancing gut barrier function and reducing bacterial translocation, and promoting anti-inflammatory and anti-pathogenic activity.^30^ Lactobacillus casei, which secretes ferrichrome metabolites, exerts anti-cancer effects by triggering apoptosis in tumor cells.^59^ Furthermore, the probiotic *Lactobacillus rhamnosus* GG (LGG) exerts anti-proliferative factors in human gastric and colonic cancer cells, with evidenced anti-inflammatory effects on the intestinal environment, and has been shown to attenuate toxic treatment-related side effects.^30,59^

Cancer and anti-cancer therapies can also impact the physiological stress response, including stress hormones such as cortisol, and the HPA axis. In healthy individuals, the stress response and the HPA axis help maintain homeostasis. However, under adverse conditions, such as injury or disease, this system can become dysregulated. Although HPA-axis dysfunction is unlikely to be a causative factor in cancer development, chronic stress does have the potential to compromise the immune system. Additionally, chronic stress has been shown to promote gut permeability, “leaky gut,” increasing potential translocation of harmful bacteria and their products into the blood stream which can induce a systemic inflammatory response.^2^ A compromised immune system in conjunction with a dysbiotic gut may, therefore, have the potential to increase one’s vulnerability to developing cancer.

For example, chronic stress, poor nutritional habits, and gut microbiota dysbiosis have been associated with development of colorectal cancer.^59^ Moreover, cancer itself, anticancer treatments and the psychosocial stress that often accompanies a cancer diagnosis may converge, subsequently leading to dysregulation of the HPA axis. Additionally, it is possible that certain treatment regimens for cancer, such as those involving corticosteroid treatments (eg, dexamethasone), have disruptive effects on HPA axis function. Cancer treatments and dysregulation of the immune system also elicit sickness behaviors (ie, a suite of organized behaviors such as lethargy, anhedonia, social withdrawal, and anhedonia), induced by systemic pro-inflammatory cytokine activity, and is associated with HPA-axis dysregulation.^54^ Probiotics have been shown to affect the HPA axis and to reduce intestinal permeability. In rats, Ait-Belgnaoui et al^60^ found that when treated with Lactobacillus farciminis, rats showed suppressed HPA axis responses to stress, which was associated with protection against compromised permeability of the intestinal barrier and decreased levels of circulating LPS. Probiotics may, therefore, contribute to protection against anti-cancer therapy toxicities by supporting immune and HPA-axis functions.

### Limitations, Gaps in Research and Future Directions

Limitations of the present review include a limited number of studies meeting the inclusion criteria, considerable heterogeneity in the types, doses and duration of interventions used, and inconsistent reporting of study characteristics between studies. These factors present challenges for drawing robust conclusions about the data.

Research on prebiotic and probiotic supplementation focused on health outcomes within cancer cohorts is in its infancy. This study revealed numerous gaps within the research, presenting an opportunity to improve not only the quality of this type of research, but the care of people affected by cancer. Notably, considerable inconsistencies in the strain, dose and treatment regimen for probiotic supplementation is an ongoing challenge. Insufficient research exists to find consensus; however, this is something to work toward.

Research suggests that patients generally have poor knowledge regarding probiotic supplementation, especially about potential adverse side effects. Ciernikova et al^61^ examined rates of use and knowledge regarding probiotics in 499 cancer patients treated in an out-patient clinic and found that 28.5% reported using probiotics, 61.3% of which reported positive effects of treatment, and only 8.5% indicating negative side effects, such as diarrhea and gas. However, up to 86.6% of patients declared no knowledge of possible risks, and indicated that their decision to take probiotics was influenced primarily by recommendations from their doctor (37.3%), relatives (23.2%), or the media (17.6%).^61^ Without sufficient knowledge, patients may be more likely to use probiotic supplements incorrectly, with potential to experience adverse reactions, or at the least gain no benefits. Future research must focus on developing tools and strategies to help educate patients on the safe and effective supplementation of probiotics, supporting their autonomy as advocates for their health.

This systematic review highlights the need for interdisciplinary collaboration to plan and conduct studies that examine both physical and psychosocial health outcomes in cancer cohorts. There exists an enormous opportunity for behavioral scientists to work collaboratively with specialists in gastroenterology, physiology, microbiology, and oncology. More controlled trials using feasibility and pilot study designs before proceeding to larger RCTs are needed, particularly for those that measure GI and psychosocial health-related outcomes in the context of a prebiotic or probiotic intervention. Future studies must begin conceptualizing the gut and brain as an interconnected system, via the microbiota-gut-brain axis, and evaluate all components accordingly. Studies can be improved by targeting both the gut, via probiotics, and the brain via behavioral intervention, such as cognitive behavioral therapy (CBT), mindfulness-based stress reduction (MBSR) or yoga, to target the microbiota-gut-brain axis within a single intervention and optimize treatment effects for participants.

## Conclusions

The most commonly used probiotic strains are from the *Lactobacillus* genus. There is some evidence for the effectiveness of probiotics in treating GI symptoms, particularly diarrhea, abdominal pain, and gas and bloating in cancer patients and survivors. Few studies have investigated the effects of prebiotics on GI and psychosocial health outcomes, reporting inconsistent improvements in select outcomes. There is limited evidence for probiotics in treating psychosocial health-related issues within cancer cohorts, however, this is likely because most studies fail to measure such outcomes where a probiotic intervention was implemented. Probiotics, when supplemented after the completion of anti-cancer therapies, may help to improve survivors QOL, in addition to some GI symptoms.

## Appendix

**Appendix A.** List of Search Terms.

Concepts	Search terms
Prebiotics and probiotics	Prebiotic* [Title/Abstract] OR prebiotics [MeSH Terms] OR probiotic* [Title/Abstract] OR probiotics [MeSH Terms] OR “psychobiotic*” [Title/Abstract] OR “gut microbiome” [Title/Abstract] OR gastrointestinal microbiome [MeSH Terms] OR “gut microbiota” [Title/Abstract] OR “fermented food*” [Title/Abstract] OR “fermented foods and beverages” [MeSH Terms] OR Yogurt [Title/Abstract; MeSH Terms] OR yoghurt [Title/Abstract] OR kefir [Title/Abstract; MeSH Terms] OR dysbiosis [Title/Abstract; MeSH Terms] OR kimchi [Title/Abstract] OR kombucha [Title/Abstract]; “kombucha tea” [MeSH Terms] OR sauerkraut [Title/Abstract] OR miso [Title/Abtract] OR “soy foods” [MeSH Terms] OR bifidobacterium [MeSH Terms] OR “Bifidobacterium animalis” [Title/Abstract] OR “Bifidobacterium longum” [Title/Abstract] OR “bifidus regularis” [Title/Abstract] OR Bifantis [Title/Abstract] OR “lactobacillus rhamnosus” [MeSH Terms] OR lactobacillus [MeSH Terms] OR “aacharomyces boulardii” [Title/Abstract] OR Saccharomyces [MeSH Terms] OR Streptococcus [MeSH Terms] OR Enterococcus [MeSH Terms] OR Escherichia [MeSH Terms] OR Bacillus [MeSH Terms] OR “Lactobaccilus helveticus” [MeSH Terms] OR “Lactobacillus casei” [MeSH Terms] OR “Lactobacillus plantarum” [MeSH Terms] OR “Lactobacillus acidophilus” [MeSH Terms] OR “Lactobacillus delbrueckii” [MeSH Terms] OR “Bifidobacterium breve” [MeSH Terms] OR “Bifidobacterium longum subspecies infantis” [MeSH terms] OR “Streptococcus salivarius” [MeSH terms] OR “Lactobacillus gasseri” [MeSH Terms]
Cancer	neoplasms[MeSH Terms] OR “cancer survivor*” [Title/Abstract] OR “cancer survivors”[MeSH Terms] OR neoplasm* [Title/Abstract] OR cancer [Title/Abstract] OR carcinoma [Title/Abstract; MeSH Terms] OR tumor* [Title/Abstract] OR tumour* [Title/Abstract] OR oncology [Title/Abstract] OR “medical oncology” [MeSH Terms] OR malignancy [Title/Abstract] OR malignant [Title/Abstract] OR chemotherapy [Title/Abstract] OR “drug therapy” [MeSH Terms] OR “antineoplastic combined chemotherapy protocols” [MeSH Terms] OR metastasis [Title/Abstract] OR neoplasm metastasis [MeSH Terms] OR metastases [Title/Abstract] OR radiotherapy [Title/Abstract; MeSH Terms] OR radiation [Title/Abstract; MeSH Terms] OR “irradiation” [Title/Abstract]) OR “gastrointestinal cancer*” [Title/Absract] OR “gastrointestinal carcinoma” [Title/Abstract] OR “gastrointestinal neoplasm*” [Title/Abstract] OR “gastrointestinal tumor*” [Title/Abstract] OR “gastrointestinal tumour*” [Title/Abstract] OR “gastrointestinal neoplasms” [MeSH Terms] OR “gastrointestinal stromal tumors” [MeSH terms]
Clinical studies	Clinical Trial [Publication Type] OR trial* [Title/Abstract] OR feasibil* [Title/Abstract] OR pilot [Title/Abstract] OR random* [Title/Abstract] OR experiment* [Title/Abstract] OR intervention [Title/Abstract] OR program* [Title/Abstract] OR group* [Title/Abstract] OR arm [Title/Abstract] OR single-arm [Title/Abstract] OR single-group [Title/Abstract] OR control* [Title/Abstract] OR comparison [Title/Abstract] OR clinical [Title/Abstract]
Psychosocial health	QOL [Title/Abstract] OR “quality of life” [Mesh terms] OR “quality of life” [Title/Abstract] OR “well-being” [Title/Abstract; MeSH terms] OR “activities of daily living” [Title/Abstract; MeSH Terms] OR “social conditions” [MeSH terms] OR “social condition*” [Title/Abstract] OR “psychosocial well-being” [Title/Abstract] OR stress [Title/Abstract] OR psychology, social [MeSH Terms] OR rejection, psychology [MeSH terms] OR “psychological adaptation” [Title/Abstract] OR psychology, behavioral [Title/Abstract] OR “social psychology” [Title/Abstract] OR “psychological distress” [Title/Abstract; MeSH Terms] OR “emotional adjustment” [Title/Abstract; MeSH terms] OR isolation [Title/Abstract] OR “mental health” [Title/Abstract; MeSH Terms] OR emotion* [Title/Abstract] OR emotions [MeSH terms] OR “emotional adjustment” [Title/Abstract] OR “holistic nursing” [MeSH terms] OR anxiety [Title/Abstract; MeSH Terms] OR “social support” [Title/Abstract; MeSH terms] OR anger [Title/Abstract] OR mood [Title/Abstract] OR social [Title/Abstract] OR “social stigma” [Title/Abstract] OR “social factor” [MeSH terms] OR “social factor*” [Title/Abstract] OR distress [Title/Abstract] OR depression [Title/Abstract; MeSH terms] OR isolation [Title/Abstract] OR “fear of cancer recurrence” [Title/Abstract] OR fatigue [Title/Abstract; MeSH Terms] OR “mental fatigue” [Title/Abstract; MeSH Terms] OR hopelessness [Title/Abstract] OR immobility [Title/Abstract] OR “psychosocial factor*” [Abstract/Title] OR “functional assessment” [Title/Abstract] OR cognition [Title/Abstract; MeSH Terms] OR “cognition disorder*” [Title/Abstract] OR “cognition disorders” [MeSH Terms] OR “cognitive decline” [Title/Abstract] OR “cognitive dysfunction” [MeSH Terms] OR pain [Title/Abstract; MeSH Terms] OR “social functioning” [Title/Abstract] OR “social behaviour” [Title/Abstract] OR “social behavior” [Title/Abstract; MeSH terms] OR “emotional well-being” [Title/Abstract]
Gastrointestinal health	Nausea [Title/Abstract; MeSH Terms] OR nauseous [Title/Abstract] OR vomiting [Title/Abstract; MeSH Terms] OR constipat* [Title/Abstract] OR constipation [MeSH Terms] OR diarrhea [Title/Abstract; MeSH Terms] OR diarrhoea [Title/Abstract] OR pain [Title/Abstract; MeSH Terms] OR “stomach pain” [Title/Abstract] OR “abdominal pain” [Title/Abstract; MeSH terms] OR gas [Title/Abstract] OR bloating [Title/Abstract] OR IBS [Title/Abstract] OR “irritable bowel syndrome” [Title/Abstract; MeSH Terms]; cramps [Title/Abstract] OR “stomach cramps” [Title/Abstract] OR “acid reflux” [Title/Abstract] OR “gastroesophageal reflux” [MeSH Terms] OR heartburn [Title/Abstract; MeSH Terms] OR GERD [Title/Abstract] OR dyspepsia [Title/Abstract; MeSH Terms] OR indigestion [Title/Abstract]
